# Diagnostic and Therapeutic Challenges of Postoperative Bronchoesophageal Fistula Leading to Acute Respiratory Distress Syndrome

**DOI:** 10.7759/cureus.81127

**Published:** 2025-03-24

**Authors:** Si Jia Lee, Bridget Ng, Suhitharan Thangavelautham

**Affiliations:** 1 Anaesthesiology and Perioperative Medicine, Singapore General Hospital, Singapore, SGP

**Keywords:** acute respiratory distress syndrome (ards), bronchoesophageal fistula (bof), esophagectomy complications, extracorporeal membrane oxygenation, ventilatory management

## Abstract

Bronchoesophageal fistula (BOF) is a rare but severe complication following Ivor-Lewis esophagectomy, often leading to aspiration pneumonia and acute respiratory distress syndrome (ARDS), creating significant diagnostic and management challenges. We report a case of a man who developed BOF 26 days postoperatively, initially diagnosed as hospital-acquired pneumonia. Rapid respiratory deterioration led to intensive care unit (ICU) admission, where persistent air leaks prompted bronchoscopic confirmation of BOF. Despite endoscopic stenting and lung-protective ventilation, severe ARDS necessitated interim veno-venous extracorporeal membrane oxygenation (vv-ECMO). Unfortunately, the patient developed multiorgan failure and succumbed after 34 days on ECMO. This case underscores the importance of early diagnosis, multidisciplinary management, and balancing ventilatory strategies to support both ARDS treatment and fistula healing. BOF should be suspected in postesophagectomy patients with respiratory symptoms, even with initially negative contrast studies, and managed promptly with customized ventilation, early fistula repair, and timely ECMO support to optimize outcomes.

## Introduction

Bronchoesophageal fistula (BOF) after Ivor-Lewis esophagectomy is an uncommon but devastating surgical complication with an incidence of 1%-4% and a mortality rate of up to 60% [1]. The pathological tract formed between the respiratory system and gastrointestinal tract causes recurrent aspiration pneumonia, which can deteriorate into acute respiratory distress syndrome (ARDS). Patients with concomitant BOF and ARDS pose several ventilatory and treatment challenges in the critical care setting because the ventilatory requirements of each condition tend to be conflicting [2]. However, literature on the management of BOF in the presence of ARDS is scarce, mainly in the form of case reports and small series. We present a case of BOF post-minimally invasive esophagectomy (MIE), complicated by ARDS, highlighting several diagnostic and management challenges faced and valuable lessons learned.

## Case presentation

Our patient, who had hypertension, hyperlipidemia, and single-vessel ischemic heart disease, underwent minimally invasive Ivor-Lewis esophagectomy for locally advanced distal esophageal adenocarcinoma (T3N0M0). He had received neoadjuvant chemoradiation (chemoradiotherapy for esophageal cancer followed by surgery study (CROSS) regime) prior to his MIE. Postoperatively on postoperative day (POD) 5, the patient had a negative routine contrast study to evaluate the anastomosis. During the Gastrografin swallow on POD 5, he was noted to have a left pneumothorax incidentally. This was conservatively managed with oxygen therapy, regular chest physiotherapy, and incentive spirometry. He remained well, was asymptomatic, and had no desaturations inpatient. The repeat chest radiograph (CXR) done prior to discharge showed resolution of the left pneumothorax. He was allowed oral intake on POD 6, and he was eating well with no swallowing issues. The patient had an uneventful recovery with no persistent coughing, no desaturation, and normal blood gas results and was discharged home well on POD 9. When seen in the clinic as part of the routine postsurgery follow-up one week after discharge, the patient was well with no complaints.

**Figure 1:**
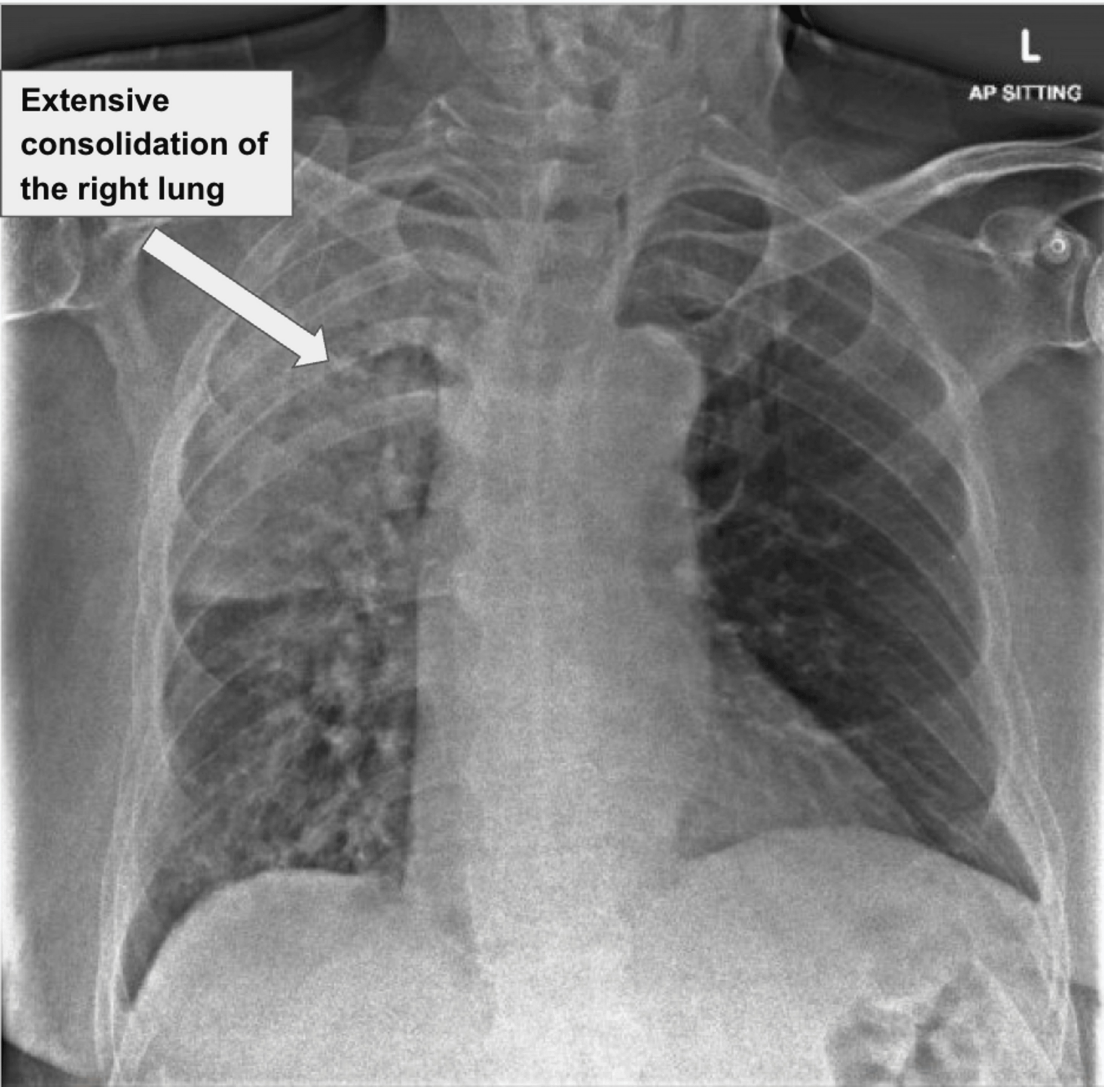
Chest radiograph demonstrating extensive consolidation of the right lung, characterized by widespread opacification and loss of normal aeration

He later presented to the emergency department (ED) on POD 26 with dyspnea, cough, and mild chest pain of two weeks’ duration. A CXR in the ED showed extensive consolidation of the right lung (Figure 1).

**Figure 2:**
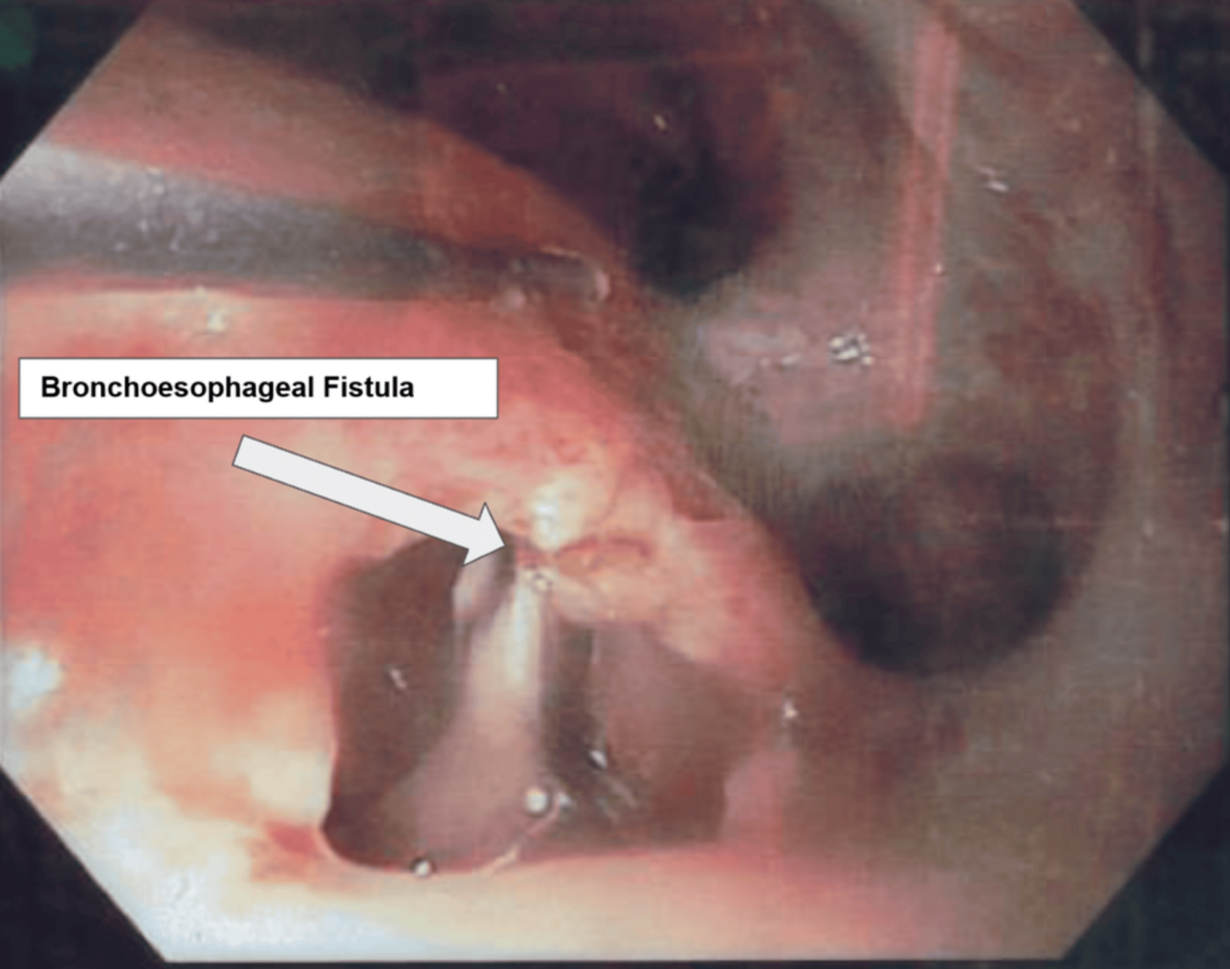
Bronchoscopy demonstrated direct communication between the bronchial lumen and the esophagus, with visible mucosal disruption at the site of the fistula

The primary upper gastrointestinal surgeon who performed the initial esophagectomy was consulted on admission as the differential diagnosis of BOF was considered. However, in view of the previous negative routine contrast study, late presentation, and the absence of pleural effusion on the CXR in the ED, the differential diagnosis of BOF was thought to be less likely. The patient was primarily treated for hospital-acquired pneumonia in view of his clinical symptoms and CXR findings. He was started on piperacillin-tazobactam and oseltamivir, and a full workup for pneumonia, including sputum gram stain, cultures, *Mycobacterium tuberculosis* ProbeTec™ (Becton Dickinson and Company, Franklin Lakes, NJ, US), urine *Legionella*, and *Streptococcus* antigen, was performed. His oxygen requirements escalated rapidly, and within 48 hours of initial presentation, he required admission to the intensive care unit (ICU) for intubation and mechanical ventilation. There were no significant findings of a distended stomach on CXR, and the abdominal X-ray (AXR) did not show large amounts of gas in the bowel lumen as well. The diagnosis of BOF was revisited and confirmed later on ICU day 5 via a bronchoscopic examination (Figure 2) following the detection of a persistent air leak on the ventilator. No repeat water-soluble contrast studies were conducted as the diagnosis was made after the bronchoscopic examination in the ICU.

**Figure 3:**
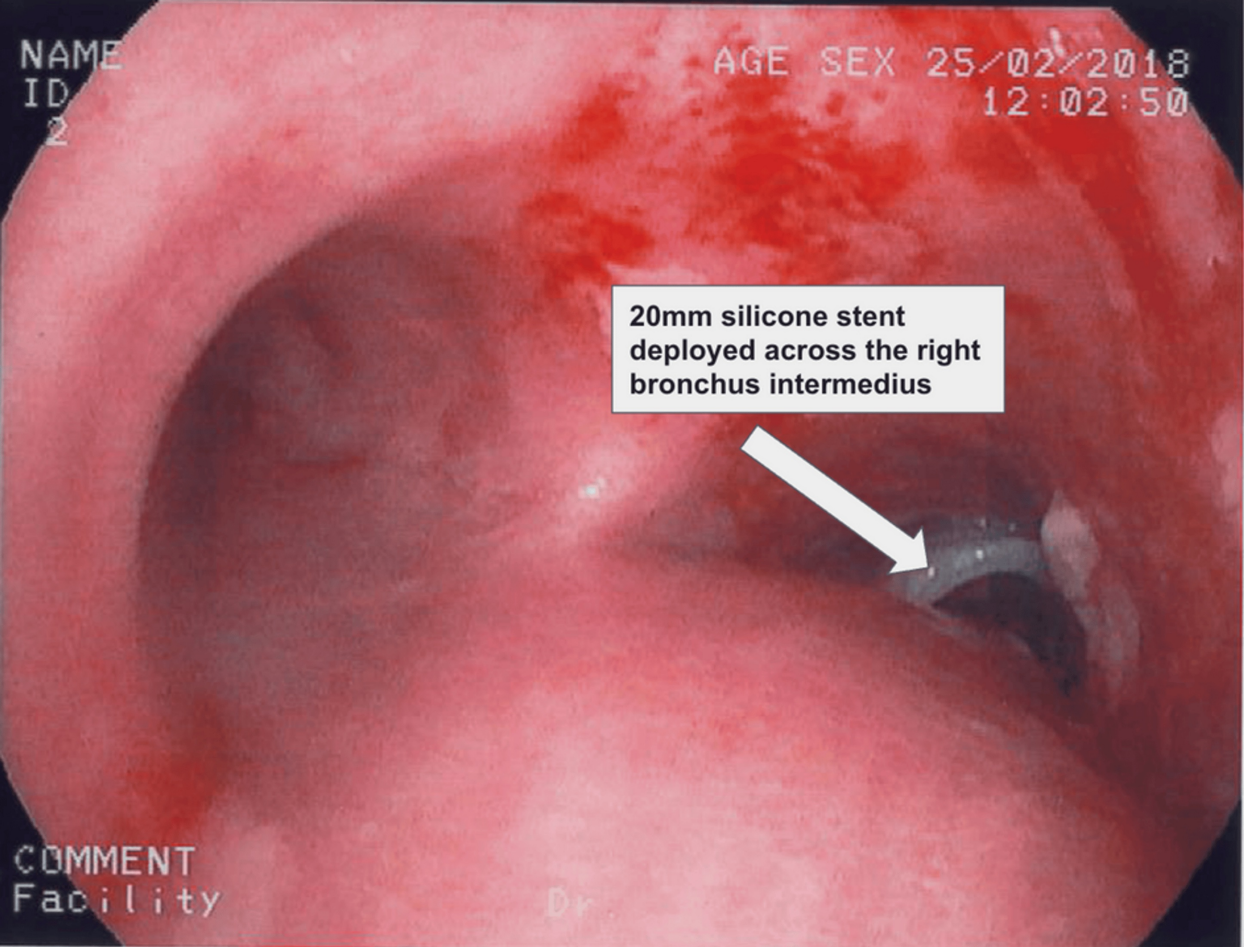
A 20-mm silicone stent deployed across the right bronchus intermedius, intentionally occluding the right upper lobe due to the fistula's proximity

An esophago-gastro-duodenoscopy (OGD) was performed on the same day, whereby an attempt to repair the anastomotic leak with clips was unsuccessful due to extensive fibrosis at the gastroesophageal anastomosis. Instead, an esophageal straight stent (Ultraflex^TM^, Boston Scientific, Marlborough, MA, US) was placed across the fistula. A 20-mm silicone stent (Figure 3) was deployed across the right bronchus intermedius, intentionally occluding the right upper lobe as the fistula was too close to its lumen. The air leak resolved completely thereafter.

In the interim, the patient developed severe ARDS (partial pressure oxygen to inspired oxygen ratio 154) and was paralyzed on ICU day six. He was paralyzed for 48 hours and ventilated on the following lung-protective ventilatory settings: volume-control mode, fraction of inspired oxygen (FiO_2_) 0.7, tidal volumes (TVs) 400 mL, and positive end-expiratory pressure (PEEP) 12 cmH_2_O. After stopping the IV paralytics, our patient was turned prone and switched to pressure support mode, allowing spontaneous breathing. The ventilator settings were as follows: FiO_2_ 0.55, pressure support 16 cmH_2_O, and PEEP 12 cmH_2_O. The patient completed a total of two prone sessions, each lasting 16 hours. During the second prone session on ICU day 12, assist volume-control mode was used, with a ventilator setting of FiO_2_ 0.45, TV 420 mL, and PEEP 12 cmH_2_O. A leak of 100-150 mL was detected. The lung isolation ventilatory strategy was considered at some point due to the high PEEP requirements, which may not be conducive to fistula healing. However, the team had concerns that the tracheal tip of the double-lumen tube may inevitably migrate and traumatize the fistula, given the fistula’s proximity to the carina.

A repeat bedside bronchoscopic examination on ICU day 12 revealed a proximal extension of the fistula, possibly due to the high ventilatory settings. A multidisciplinary meeting involving the intensivist, respiratory physician, and surgeon was held, and the consensus was made to perform a rigid bronchoscopic insertion of a Y stent in the emergency operating theater. Our patient had a worsening alveolar-arterial (A-a) gradient since admission as well and was deemed unlikely to be able to tolerate one-lung ventilation (OLV). His PAO_2_ - PaO_2_ (A-a) gradient on admission was 135 mmHg, at the point of paralysis was 385 mmHg, and after turning prone was 242 mmHg. In view of worsening air leak (increased to 300 mL) and concomitant ARDS, a joint decision was made to prophylactically initiate veno-venous extracorporeal membrane oxygenation (vv-ECMO) prior to but at the same seating of the bronchial stenting. The A-a gradient of the patient was 241 mmHg prior to initiating vv-ECMO. The Y stent was satisfactorily deployed over the right main bronchus, occluding the entire length of the fistula.

He was continued on vv-ECMO support for the next four weeks with minimal air leak, but his respiratory condition fluctuated with no clear improvement. He subsequently developed fungemia with septic shock and multiorgan failure including acute kidney injury requiring continuous renal replacement therapy. In view of medical futility, vv-ECMO was withdrawn after 34 days and the patient died 48 hours later. The decision to withdraw vv-ECMO was a combined one made between the intensivist, primary surgeon, respiratory physician, and family.

## Discussion

Despite the severity of post-BOF, there are currently no established consensus guidelines for its diagnosis and management. In this case, a delay in diagnosis was considered a significant factor contributing to the patient's poor outcome. Such delays are not uncommon, as demonstrated in this report. This case highlights the challenges in the early identification of BOF, which is often difficult to detect at the time of admission and may only become evident as the patient's condition deteriorates.

Anastomotic leaks are generally considered to be the precursor for acquired BOF following esophagectomy [3]. However, the routine POD 5 negative surveillance water-soluble contrast study the patient had prior to this readmission and the apparent absence of an air leak during the initial mechanical ventilation were factors that likely detracted the team from the diagnosis of BOF. In addition, our patient’s presentation on POD 26 is relatively late for BOF complications. In a retrospective study, 11 out of 13 of their patients with postesophagectomy anastomotic leak presented within two weeks of surgery [4].

In hindsight, the index of suspicion for BOF needs to remain high in all postesophagectomy patients presenting with pneumonia, regardless of early negative contrast studies. From the outset, patients presenting with pneumonia after esophagectomy should have a repeat contrast study performed. The threshold for ordering additional investigations such as a computed tomography of the chest or endoscopy should be low. This will ensure early detection of BOF and expeditious interventions.

Our institution does not have a protocol for the management of BOFs. Based on our experience from this case, we conclude that the approach to the management of BOF with ARDS should be multipronged and can be distilled into three key strategies: customized ventilatory strategies encompassing low airway pressures and lung isolation to facilitate fistula healing, early repair of the fistula via double stenting or surgery, and early consideration and implementation of ECMO as an interim modality to allow lung rest.

Ventilatory settings that are required to adequately oxygenate patients with ARDS are often detrimental to the healing of fistulas. In the presence of a bronchial fistula, we aim to promote healing by minimizing the distending pressures imposed during positive pressure ventilation (PPV). This includes ensuring both a low driving pressure and low PEEP, the former best achieved with spontaneous ventilation modes. However, in severe ARDS, lung compliance is frequently poor, necessitating a relatively high driving pressure to achieve adequate TVs of 4-6 mL/kg of predicted body weight. Extensive atelectasis and consolidation also warrant a high PEEP to recruit alveolar units to optimize shunt fraction and overall oxygenation [5]. Paralysis, another treatment modality recommended for severe ARDS, requires PPV, which is also less ideal for fistula healing [6]. Our patient was ventilated on a PEEP of 12-14 cmH_2_O, and we tried to allow spontaneous breathing as far as possible, including during periods of prone positioning. Unfortunately, ventilating our patient based on the above strategies was a significant limitation because the fistula could have consequently been subjected to high distending pressures (PEEP and inspiratory pressures), which likely impeded its healing and contributed to its enlargement.

One solution to the problem of conflicting ventilatory requirements in co-existing BOF and ARDS is to perform lung isolation with OLV or differential ventilation. Here, the lung with the fistula is “rested” on minimal ventilatory settings, while the non-fistula lung is ventilated according to ARDS settings [6]. We initially rejected the lung isolation strategy as the tip double-lumen tube was deemed potentially injurious to the fistula by virtue of the fistula’s proximal position in the right main bronchus. However, in hindsight, lung isolation could have been alternatively achieved by performing a bronchoscopic-directed left bronchial intubation and left lung ventilation via a single lumen tube. We subsequently lost the window of opportunity for OLV when severe ARDS set in and our patient’s A-a gradient increased by two- to threefold (at the point of paralysis was 385 mmHg and after two sessions of prone positioning was 242 mmHg). Prior to the initiation of vv-ECMO, his A-a gradient was 241 mmHg.

Early diagnosis and repair of the fistula and effective leak management are crucial for optimal outcomes. In our case, the primary upper gastrointestinal surgeons were consulted immediately upon readmission. Furthermore, when it comes to the endoscopic management of BOF, a multidisciplinary approach should be sought between the endoscopist and bronchoscopist following careful evaluation of the anatomy. Double stenting is likely to play an important role in fistula healing, especially so in cases requiring high ventilatory settings or when OLV cannot be established. Some evidence exists suggesting that double stenting conferred higher rates of complete fistula closure and possibly improved quality of life and survival [7,8]. In both series reported by Schweigert et al. and Lambertz et al., majority of their patients received either double stenting or surgical repair [3,4]. Among those who received double stenting, Y stent was the choice of bronchial stent. It is thought that BOFs close to the carina are likely to benefit from a Y stent or an appropriately sized silicon stent [9]. In our case, although we performed double stenting, a silicon straight bronchial stent was used initially as the Y stent was unavailable. The latter was deployed only six days later when a ventilatory leak had recurred. It is possible that during this period, the seal provided by the silicon straight stent was suboptimal, subjecting the bronchial fistula to the rigors of high airway pressures, resulting in extension. A recent review article suggested 3D-printed airway stents as a potential future development. This could allow for customized fitting and prevent the potential risks of paradoxical fistula enlargements in ill-fitting stents [10].

Lastly, ECMO needs to be considered early as an interim modality in cases of BOF complicated by ARDS. ECMO allows for lung rest via the implementation of ultraprotective lung ventilatory strategies (2-3 mL/kg of predicted body weight), creating a low-pressure environment conducive to fistula healing. It also mitigates the injurious effects of mechanical ventilation and promotes healing of the lung parenchyma [11]. Several case reports have demonstrated good outcomes with the use of vv-ECMO in the context of BOF and ARDS [12-14]. One case series of four patients with surgical fistulas and ARDS described the use of pumpless extracorporeal carbon dioxide removal as a less invasive and efficient bridging therapy compared to vv-ECMO [15]. In our patient, ECMO was sought as a last resort and activated only as a backup for anticipated peri-procedural ventilation failure. Because ECMO buys time for fistula healing while providing adequate oxygenation, in cases where definitive fistula repair cannot be achieved, it can serve a pivotal role and should be considered early so as to capture the window for fistula healing.

## Conclusions

This case highlights the necessity of maintaining a high index of suspicion for BOF in postesophagectomy patients presenting with pneumonia-like symptoms, even if initial contrast studies are negative and the timing of presentation is beyond the typical clinical window for surgical complications. Prompt and accurate diagnosis through endoscopic or radiological assessment is critical to preventing severe complications. Ventilatory management poses unique challenges in BOF with ARDS, requiring a delicate balance between lung-protective strategies for ARDS and minimizing airway pressures to facilitate fistula healing. Lung isolation as a ventilatory strategy should also be considered early to protect the fistula from PPV. When conventional ventilation fails, early initiation of ECMO can provide crucial respiratory support and create conditions favorable for fistula closure, further emphasizing the need for timely intervention and a multidisciplinary treatment approach.

With regard to the current interventional options available to address the fistula, double stenting appears to be the most effective strategy, while the use of Y stents or customized airway stents may offer superior seal and reduce the risk of fistula progression. Emerging technologies such as 3D-printed stents hold great potential in facilitating this specific clinical outcome.
